# Examining the trade-offs in clean energy provision: Focusing on the relationship between technology transfer, renewable energy, industrial growth, and carbon footprint reduction

**DOI:** 10.1016/j.heliyon.2023.e20271

**Published:** 2023-09-22

**Authors:** Qiyun Zhou, Jianpeng Wu, Muhammad Imran, Abdelmohsen A. Nassani, Rima H. Binsaeed, Khalid Zaman

**Affiliations:** a Economics Management College, Foshan Polytechnic, Foshan 528137, China; b Department of Economics, The University of Haripur, Haripur Khyber Pakhtunkhwa 22620, Pakistan; c Department of Management, College of Business Administration, King Saud University, P.O. Box 71115, Riyadh 11587, Saudi Arabia

**Keywords:** Technology transfer, Regional economic integration, Carbon emissions, Renewable energy demand, Trade openness, Differenced GMM estimator

## Abstract

Sustainable energy mitigates climate change by reducing reliance on coal and oil for power generation, curbing global warming. It addresses environmental concerns and yields economic benefits—reduced fossil fuel dependence, financial inclusion, productive employment, and economic development. This research examines the impact of regional economic integration on environmental sustainability in 39 high-income European and Central Asian (ECA) nations from 2017 to 2021. Specifically, the study analyzes the influence of green energy demand, technological transfers, and trade openness on carbon emissions. The study employed various estimators, namely, a two-step Generalized Method of Moments (GMM) estimation, quantile regression, and the cointegration panel approach. These estimators were utilized to capture different aspects and dynamics of the research variables. The study finds that regional green programs and trade agreements effectively reduce carbon emissions, while technological advances and industrial output tend to raise them. Granger causality analyses reveal that emissions-led regional development, technical innovation, and trade openness are interconnected factors, and the deployment of renewable energy contributes to carbon emissions. The inter-temporal analysis suggests that regional economic integration factors will likely impact carbon emissions in the following decade. These findings support neoclassical growth theory, new institutional economics, and ecological modernization theory. Developing renewable energy sources in the region can minimize energy price fluctuations, strengthen energy security, and align with the carbon neutrality agenda. This research emphasizes the need for sustainable energy strategies and regional cooperation to foster a greener and more sustainable future.

## 1 Introduction

The regional economic partnership may mitigate climate change's effects. Regional commercial deals may help governments collaborate to reduce GHG emissions, increase energy efficiency, and promote alternatives to fossil fuels [1]. Emissions-cap trading in the EU has reduced CO2 emissions. Economic convergence across areas may help spread carbon-friendly products and practices and create climate change strategies [2]. Integrating the regional economy may enable joint action to address complicated climate change issues [3]. The sharing economy safeguards social and environmental goals by creating a novel framework for reducing pollution and waste and meeting many eco-friendly objectives [4]. The operations of the circular economy tend toward clean and green production, necessitating a technical application and knowledge that allows for cutting down on economic waste of resources and safeguarding economic and environmental sustainability [5]. Maintaining and exchanging the flow of products, services, physical and human capital, information, and skills is essential to the success of regional and worldwide economic integration, which is facilitated by developing a shared physical and institutional infrastructure [6,7]. GHG stabilization and increased absorption in the environment at low levels appropriate for the climate system are the primary goals of environmental stakeholders and institutions [8,9]. The world's economies have primarily concluded that immediate action on climate change, backed by more substantial resources and enthusiasm, is necessary to fulfill the objectives of the Paris Agreement [[10], [11], [12]].

ECA nations are vital to global environmental sustainability. Europe emitted 10% of global GHGs and pledged a 40% reduction by 2030 [13]. Wind power provided 22% of the EU's renewable energy in 2020, second only to solar [14]. The Central Asia Regional Economic Cooperation (CAREC) initiative seeks to increase the region's renewable energy (REC) capacity by using its wind and hydropower potential [15]. The Central Asia-EU Energy agreement is a viable project to improve REC and energy efficiency. These activities illustrate the need for regional integration and cooperation in solving sustainability issues [16,17]. Municipalities, economic regions, businesses, and investors must act for clean initiatives for economic activities. One should help implement the Paris Agreement's goals of reducing environmental emissions to keep the average global temperature at 1.2 °C, higher in industrial and high-income countries [18,19]. Kumar et al. [20] concluded that the COVID-19 pandemic globally threatens the health, the environment, energy, and the economy. COVID-19 slows the economy and human activity, reducing GHG emissions and atmospheric CO2. Regional integration affects urban expansion, policy environment, climate change, and environmental sustainability [21]. The main issues are strengthening institutional capacity and organizational management sequencing to foresee changes in industrial output, other pollutants, and emissions and enacting strong environmental laws. Onifade et al. [22] suggested that clean advances in environment and regulation and well-crafted economic integration plans may attract more sustainable environments and fewer climate changes locally and globally. Due to population ageing and economic globalization, climate and sustainability issues are difficult to solve [23,24].

### 1.1 Essential factors in successful expansion plans

The study contributes to the scientific understanding of regional economic integration and its impact on environmental sustainability. It introduces three key factors—technology transfer, green energy supply, and trade openness—related to regional economic integration, which have been overlooked in previous research that primarily focused on specific aspects like supply chain processes, alternative energy sources, and technological innovation. By considering these factors together, the study provides a comprehensive perspective for developing long-term strategies for regional growth and environmental sustainability. The study breakthrough in three ways: first, it uses three key factors related to regional economic integration, such as technology transfer, green energy supply, and trade openness, where previous research has focused primarily on the supply chain process [25,26], alternative energy sources [27,28], the circular economy [5], technological innovation [4], and international collaboration [5]. If one is disregarded, it becomes difficult to create long-term strategies for regional growth. Second, regional efforts for cleaner production via sustainable development were derived from the study's utilization of industrial value added and economic growth. The past research employed such linked elements in various areas or specific nation cases [29], which leaves the possibility for the approach to work for high-income countries in ECA region. Finally, the research introduces a dummy variable with value one for the 2019–2021 dataset to evaluate the effect of the pandemic on environmental quality; this allows for more solid green conclusions, given the COVID-19 pandemic's inherent fragility.

This research looks at the dynamics in Europe and Central Asia (ECA) to see whether sustainable energy may help combat climate change and stimulate economic development. The study aims to examine the complex dynamics between economic growth and ecological stability in different regions. The study does this by answering three critical questions. The first part of the research looks at the synergistic effects of technological progress, economies of scale, and regional industrial ecology on carbon emission reductions. Second, it explores how renewable energy consumption contributes to decarbonization initiatives. Finally, the study investigates the potential consequences of commercial growth on the global temperature balance, considering emissions and climate change. The answers to these questions will provide light on how to shape a cleaner future by bringing economic growth into harmony with ecological balance.

### 1.2 Study's investigative questions

Three questions based on the above information drive our research. First, do regional industrial ecology, technical progress, and economies of scale lessen carbon emissions? The research issue asks whether regional industry, technology, and economies of scale can reduce carbon emissions. A stronger industrial foundation, superior technology, and economies of scale may improve efficiency and reduce emissions. Second, does REC help achieve decarbonization? The study explores how switching to REC sources reduces carbon emissions. REC sources produce fewer emissions than fossil fuels and can help mitigate climate change [30]. Third, how much does commercial expansion risk the global's average temperature? The question examines how business growth may affect global temperature. Commercial expansion may raise GHG emissions and global warming. Understanding the environmental implications of economic development and finding ways to reduce them is crucial [31]. Such difficulties need long-term policy formulations across states to foster economic integration.

### 1.3 Study's focus and direction

Regional economic integration is examined in a panel of ECA countries to mitigate carbon emissions and preserve long-term development. Specific goals are.

I. To explore how regional technology transfer, industrial ecology, and economic growth affect green development

Sharing innovations across areas may dramatically impact sustainable development and environmental impacts [32]. Clean technology transfer from industrialized to developing nations reduces carbon emissions and boosts the local economy. Industrial ecology promotes corporate integration with local ecosystems, waste reduction, and resource efficiency [33]. A growing economy may help sustainable development and environmental protection, requiring policies and strategies to align economic growth with sustainable development [34].

II To evaluate regional green energy efforts for carbon reduction

Regional green energy projects may boost sustainable development and carbon reduction. REC is a clean, dependable alternative to fossil fuels [35]. Local and regional governments, businesses, and civil society are also crucial to green energy initiatives [36]. Regional green energy initiatives may require additional money, infrastructure, and laws to grow. Thus, regional clean energy policies and plans help achieve the decarbonization agenda [33].

III Assessing how regional trade openness during the COVID-19 epidemic affected environmental quality

Regional trade may help or deteriorate the environment. Commerce may boost the economy but also threaten the ecology and biodiversity [37]. COVID-19 has slowed business in several locations. The outbreak has highlighted the need for regional self-sufficiency and local production to promote sustainability and minimize carbon emissions [38,39]. Thus, regional trade openness should be assessed in light of the COVID-19 pandemic, and sustainable trading practices should be encouraged [40].

The urgent need to address climate change and its attendant environmental and economic effects is central to the study's justification. Relying less on fossil fuels like coal and oil to generate electricity is a big step toward decreasing GHG emissions and helping slow climate change. One of the greatest concerns of our day is global warming, which may be significantly mitigated if the world switches to renewable energy sources. Sustainable energy is acknowledged in this research as providing both environmental advantages and significant financial ones. Improved energy security and less exposure to price swings in the global energy market result from reducing reliance on fossil fuels. This promotes economic growth by establishing a reliable energy supply and increasing energy diversity.

In contrast to other studies, this study examines how high-income ECA countries benefit from regional economic integration in terms of environmental sustainability. While other studies may have looked at the topic of sustainable energy or environmental legislation, this one focuses specifically on economic integration's impact on these vital sustainability variables. This study adds novel insights to inform policy-making and build greener, more resilient economies by illuminating the connection between economic integration and sustainable energy practices.

This research is the first to analyze how economic integration in the European Union and Central Asia has affected environmental sustainability in countries with high per capita incomes. Sustainable energy techniques, as are the benefits of regional green initiatives and trade agreements on lowering carbon emissions, are brought to light. Nevertheless, it also shows possible problems, such as how technological progress and industrial activity might affect pollution. The research highlights the interdependence of these elements and provides forecasts of their future influence, all in keeping with the aims of achieving carbon neutrality. This study underlines the need for cooperative regional efforts and long-term planning for a greener future.

The decision to choose the study area and time period from 2017 to 2021 was based on several important factors, ensuring the relevance and comprehensiveness of the study. Firstly, this time frame allows for an analysis of recent developments in the field of regional economic integration and its impact on environmental sustainability in the ECA region. We focus on recent years to present a glimpse of regional economic integration, green policies, and REC advances. This is crucial due to the global nature of environmental concerns and the necessity for timely research to influence policy choices. Second, these years correlate with global events and policy moves that affected the energy business. Several regional initiatives, trade pacts, and other sustainable energy measures are being established. The success of these initiatives and policies in promoting environmental sustainability in the ECA area may be evaluated by focusing on the period under consideration. Moreover, utilizing a consistent time period across the 39 high-income ECA nations allows for meaningful comparisons and a comprehensive analysis of regional dynamics within a relatively homogenous group. By examining a relatively homogenous set of countries, we can control for certain external factors and focus on the specific impact of regional economic integration on environmental sustainability. This ensures the validity and robustness of our findings and contributes to a deeper understanding of the relationship between economic integration and environmental outcomes in the ECA region.

The aforementioned goals would be accomplished by utilizing a two-step differenced GMM estimator, which gets over serial correlation and endogenous problems. Additionally, the Granger panel causality test and the innovation accounting matrix aid in evaluating cause-and-effect relationships and predicting estimates across time.

## 2 Literature review

The ecological modernization hypothesis suggests that technological advancement and cultural changes may help modern civilizations achieve economic prosperity and environmental sustainability [41]. The study suggests that green energy and greener manufacturing procedures reduce carbon emissions. The study also supports the new institutional economics theory that governmental, corporate, and non-governmental institutions influence economic and ecological outcomes [42,43]. The study's emphasis on regional economic integration as a key to lowering global carbon emissions suggests that institutions may help solve environmental problems. This finding also affects the neoclassical growth theory, which states that rising economies may harm the environment, but efficiency and technology may mitigate these effects [44]. Research supports this hypothesis by emphasizing the need to transfer technology and employ green energy to minimize carbon emissions and the environmental impact of economic growth.

A comprehensive review of the literature reveals a diverse range of notable studies conducted across various countries and time periods, shedding light on the multifaceted aspects of environmental sustainability and its connection with green initiatives and policies. Hessevik [45] focuses on Norway's sustainable shipping networks from 2008 to 2020, underscoring their pivotal role in achieving climate neutrality through carbon-neutral sea transportation. This study highlights how such green shipping networks contribute significantly to reducing carbon emissions and promoting eco-friendly transportation methods. In a contextual study involving three countries, Jia [46] emphasizes the importance of green financing instruments like green bonds and innovation in minimizing carbon emissions while fostering economic growth. Understanding the impact of financial mechanisms on environmentally responsible projects becomes crucial in paving the way for sustainable development. Tanweer et al. [47] examined how accelerator programs affect organizational innovation performance, emphasizing the importance of peers, mentors, and investors. Mentors and investors helped firms succeed in accelerator programs, but peers needed more effect. These findings demonstrate that Pakistani accelerator courses boost corporate performance. Khan [9] suggested empowering women may boost Pakistan's environmental sustainability. Results revealed that more women in the workforce may reduce carbon emissions. Despite improvements in women's rights and literacy, economic and industrial restrictions prevent the country from reducing its carbon impact. Aqib & Zaman [19] examined how human capital affects economic growth and environmental sustainability. Eco-friendly R&D was recommended to minimize carbon emissions. A green development strategy demands additional money for schools, hospitals, and the employment market. Turning our attention to India, Song et al. [48] take a forward-looking approach, simulating strategies for 2050 using current data points and focusing on green hydrogen. The study advocates for the widespread adoption of green hydrogen as a key solution to reduce energy costs and achieve zero carbon emissions in the electrical and industrial sectors. In a broader regional context, Sahoo et al. [49] conduct an extensive analysis of 14 Asian countries from 1990 to 2018, highlighting the significance of sustainable technical advancements and clean energy sources in curbing human-caused greenhouse gas emissions. These findings underline the potential of technological innovations in driving carbon abatement strategies in diverse economies. Acheampong et al. [50] examined succession planning and organizational longevity. The study revealed that succession preparation is crucial to a business's long-term health. The study emphasized the importance of transferring formal knowledge and preserving institutional retention through succession planning. Additionally, career development was highlighted as a crucial factor in meeting succession requirements and ensuring the organization's existence. Khan & Imran [24] focused on ECA nations in their 1990–2021 assessment of CO2 emissions. The negative correlation between per capita income and CO2 emissions validated the inverted N-shaped EKC linkage. Green power and business value added decreased CO2 emissions, whereas the prevalence of people increased them. FDI reduced carbon emissions slightly. The study's results may help policymakers create realistic CO2 reduction goals. Shifting our focus to Japan, Cheng & Lee [51] delve into sustainable hydrogen strategies, examining the role of carbon pricing, certifications, technological improvements, and coal phase-out timelines in enhancing air quality. The study underscores how a comprehensive approach to hydrogen solutions can bolster environmental quality. On the African continent, the study by Udeagha & Ngepah [52] scrutinizes South Africa's progress from 1960 to 2020, with a focus on clean technology, green energy, and institutional performance. This research emphasizes the importance of scientific collaborations and stringent legislation in promoting green development in the region. Moving to China, Lu et al. [53] offer insights from 1990 to 2019, highlighting the positive impact of green financing on governmental and private energy infrastructure investment. This investment-oriented approach has contributed to reduced carbon emissions and improved energy efficiency in the country. Meanwhile, studies by You et al. [54], Guo et al. [55], Pata et al. [56], and Yu et al. [57] investigate the influence of international technical collaborations, financial inclusion, sustainable commercial activities, and various other green initiatives on environmental sustainability across different countries and regions. These diverse studies collectively contribute to the understanding of the complex interactions between economic activities, policy measures, and environmental outcomes. The high pace of growth in the Kingdom of Bahrain has led to a corresponding rise in energy consumption, which Alam [58] investigates. The research shows that environmental degradation harms commerce and economic development over the long run. This information may help policymakers create policies that protect the environment, promote renewable energy, and boost the economy. The goals of India to improve air quality are analyzed by Alam et al. [59]. Finding a non-linear relationship between renewable energy and air quality, the research estimates that the sweet spot for renewable energy utilization is 45.75%. In both the short and long term, urbanization degrades air quality. These results inform policy suggestions in line with India's goals for a cleaner future.

**Fig. 1:**
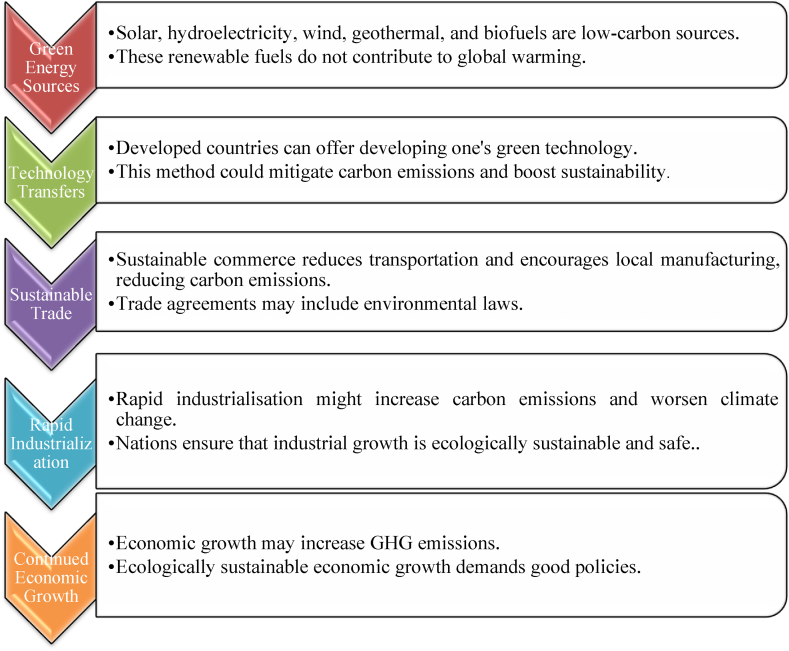
Strategies for lowering carbon emissions: A Visual Overview.

Source: Author's extract based on literature review.

Gharib et al. [60] examined how green intellectual capital affects Oman's ecology. Their results show that green structural capital affects ecological sustainability more than green human or relational capital. Green structure-based capital improves environmental sustainability in enterprises, according to a study. Nazir [61] examined how globalization, monetary growth, human capital, and carbon emissions affect renewable fuel adoption in Pakistan. The study concluded that globalization promotes green but financial products, human assets, and carbon emissions work against it. The information recommends policies to increase REC and minimize pollution in Pakistan. This study is needed to address a policy problem of long-term ecological survival and cross-border economic cooperation. This study answers a crucial topic for policymakers and stakeholders seeking sustainable development and environmental solutions: How does regional economic integration affect carbon mitigation? The research seeks to identify carbon emission growth drivers and highlight the importance of green energy initiatives, sustainable technology transfers, and industrial emission reductions. The study's sample is acceptable because the study addresses an issue of particular interest in locations where economic integration and environmental sustainability are governmental priorities. The study illuminates the relationship between economic development, regional integration, and ecological sustainability, making its results and strategies practical. Fig. 1 summarizes the literature review findings for ready reference.

The research combined literature studies from many countries and historical eras to better understand climate change policies and green activities. Carbon pricing, renewables, and carbon-neutral transportation networks are researched. This study suggests green policies and legislation to minimize carbon emissions and promote sustainable development. Hessevik [45] suggests that green shipping networks and carbon-neutral marine transportation may assist in achieving the former. Jia [46] claims that green financing can boost economic development and reduce carbon emissions. Some research disagrees. Song et al. [48] suggest employing green hydrogen to reduce energy prices and reach zero carbon emissions, whereas Sahoo et al. [49] advocate for alternate energy sources and economical carbon abatement. Various methods must be used to identify which green initiatives and policies are most helpful. Institutional effectiveness, technological cooperation, and economic growth all promote green development. Guo et al. [55] claim that economic growth increases energy efficiency, allowing businesses to switch to greener production, whereas Udeagha & Ngepah [52] indicate that partnerships and legislation aid green development. Studies provide climate change policies and eco-friendly measures. The literature offers some common ground, but diverse views need additional study. Integrating and comparing many studies, the study addresses the research gap and gives insights that may inform future policy decisions. The following research Hypotheses has been testes referring to the earlier literature, i.e..

H1 Regional technology transfer, industrial ecology, and economic growth positively influence green development.

This hypothesis suggests that as regions engage in technology transfer, adopt industrial ecology practices, and experience economic growth, it will lead to a higher level of green development. The hypothesis assumes that these factors contribute to the implementation of sustainable practices and the adoption of environmentally friendly technologies, resulting in improved environmental outcomes.

H2 Regional green energy efforts contribute to carbon reduction.

This hypothesis posits that regions that actively pursue and invest in green energy initiatives will experience a reduction in carbon emissions. It assumes that the implementation of REC sources, energy efficiency measures, and other green energy practices will lead to a decrease in carbon footprint and contribute to mitigating climate change.

H3 Regional trade openness has had an impact on environmental quality.

This hypothesis suggests that the level of regional trade openness during the COVID-19 pandemic has influenced environmental quality. It assumes that increased trade openness may have led to changes in production and consumption patterns, affecting pollution levels and other environmental indicators. The hypothesis aims to assess the relationship between trade openness and environmental quality during a period of significant disruptions caused by the pandemic.

### 2.1 Research Gap(s) and contribution of the study

The research makes several significant advances in studying environmental sustainability and regional cooperation. Previous research has focused on analyzing the cost of pollution abatement in individual countries and the correlation between economic growth and emissions [62,63]. However, this new study expands on that by considering regional economic integration as a critical factor in reducing global carbon emissions. Second, the research investigates the potential of technological exchange between ECA nations to reduce global carbon emissions. Since earlier research has yet to thoroughly investigate the connection between industrial activities in various nations and their direct and indirect influence on global emissions [[64], [65], [66]], this work represents a significant advancement in the field. Third, unlike prior studies that have solely looked at pollution mitigation techniques [67,68], this one considers the effect of REC, industrialization, and the COVID-19 epidemic on the decarbonization endeavour. Green energy deployment, cleaner manufacturing technology adoption, and disease prevention and control techniques are used in the research to measure the success of the decarbonization agenda.

## 3 Theoretical framework

Neoclassical Growth Theory holds wealth and pollution increase together [69]. Growing economies may increase pollution and GHG emissions, and technical innovation and efficiency may offset these effects and make economic development more sustainable. Development may occur without harming the environment if policies and technology investments are undertaken [70]. The study's main objective is to examine how green energy and technology may decrease carbon emissions and how regional economic integration affects environmental sustainability.

Institutions, not individuals, determine economic and ecological outcomes, according to New Institutional Economics (NIE) [71]. NIE emphasizes understanding how government, business, and NGO incentives and limits impact the economic decision. It asserts that such organizations' rules and regulations may significantly affect economic participants' behaviours and the economy's efficiency [72,73]. NIE emphasizes strong institutions' need to encourage firms to become green and enforce environmental laws. The theory also suggests considering trade-offs between economic growth and environmental conservation while building these institutions to maximize society's well-being [74].

Ecological modernization theorists believe investing in green technology research may boost economies and protect natural resources [75]. Economic development and environmental conservation are complementary in this strategy. By sponsoring environmental and green technology ventures, society may get greener. Ecological modernism and sustainable behaviour are the responsibility of all levels of society, not just governments and companies. The notion holds that society may promote economic progress and environmental protection [76].

## 4 Materials and methods

The study selected a panel of 39 high and upper-middle-income ECA countries, namely; “Albania, Andorra, Armenia, Austria, Azerbaijan, Belarus, Belgium, Bosnia and Herzegovina, Bulgaria, Croatia, Cyprus, Czechia, Denmark, Estonia, Finland, France, Georgia, Germany, Greece, Hungary, Iceland, Ireland, Italy, Kazakhstan, Latvia, Lithuania, Luxembourg, Montenegro, Netherlands, Norway, Poland, Romania, Russian Federation, Serbia, Spain, Sweden, Switzerland, Turkiye, and United Kingdom”. The data is collected from the World Bank [77] database for 2017 to 2021. The following variables used for analysis.

I. Carbon emissions (denoted by CO2, metric tons per capita) served as regressand. Carbon emissions are a key indicator of environmental impact and serve as a proxy for environmental sustainability.
II. Regional economic integration served as regressors of the study and was measured by the following key variables:
  - **Technology transfer (denoted by TECT, total residents' patent applications):** It represents the level of technological innovation and knowledge sharing within a region. It is expected that higher levels of technology transfer will lead to more sustainable practices and lower carbon emissions.
  - **Trade openness (denoted by TOP, % of GDP):** It represents the extent to which a region engages in international trade and openness to global markets. Trade openness can affect environmental outcomes, as increased trade activities may lead to changes in production and consumption patterns, influencing carbon emissions.
  - **Renewable energy consumption (denoted by REC, %of total energy use):** It represents the proportion of energy derived from renewable sources within a region. Higher levels of REC are expected to contribute to carbon emissions reduction, as REC is generally considered to be more environmentally friendly.
III. Controlled variables are as follows:
  - **Industrial value added (denoted by IND, % of GDP):** Industrial operations may significantly affect carbon emissions, especially in carbon-intensive sectors.
  - Economic growth (denoted by RGDP, constant 2015 US$ million): Economic development increases energy consumption and emissions, which may affect GHG levels.
  - **COVID-19 dummy:** This variable is a binary variable that takes the value of 1 for the years 2019–2021 and 0 for the years 2017 and 2018. It serves as a control variable to account for the potential impact on carbon emissions. The pandemic may have caused disruptions in economic activities and mobility, which could have influenced carbon emissions levels.

Studying carbon emission causes underpins the present model. Innovation, renewables, economic expansion, and freer trade reduce carbon emissions' environmental impacts. Carbon emissions also harm the ecology. GHG-emitting business activities cause these. Critical technology transfer will help spread innovative and clean technologies. Transferring renewable and clean technology to underdeveloped nations may help fight climate change. Reducing carbon emissions depends on renewables replacing fossil fuels. Renewables for GHG reduction are getting more affordable. Industrial output, a significant polluter, may rise. Thus, value added's influence on pollution in diverse industries must be examined. Growth increases energy demand and carbon emissions. Higher living standards may lower carbon emissions by stimulating energy efficiency innovation and greener technology adoption. Finally, international commerce may reduce carbon emissions by increasing access to cleaner technology, renewables, and knowledge sharing. The study aims to illuminate these elements' effects on carbon emissions to inform policies and initiatives to reduce emissions and ameliorate climate change.

The advancement of econometric techniques has led to new discussions on cross-panel techniques, such as developing cross-panel unit root tests. These tests consider a country's internal and external shocks from other countries, offering a fresh approach to empirical investigation [78]. The research used differenced panel GMM estimator. Estimating the issue of independent variables connected with the error term is made possible by the dynamic model, and the supplied estimator also controls the endogeneity issue. The Arellano & Bond [79] estimator identifies the existence of first and second-order autocorrelation and the validity of the GMM estimators. Equation (1) describes a straightforward dynamic panel data procedure:

(1) $${y_{\text{it}}=}_{.}{ɸy}_{i,t-i}+\mu_{i}+s_{\text{it}},\text{whereas};\;i=1,\ldots .N;\;t=1,\ldots .T$$

Under the following assumptions.

(i) $s_{\text{it}}$ are i.i.d across time and cross-section of ***μ***_i_ and *y*_i1_ with *E*(s_i*t*_) = 0, Var(s_i*t*_) = ***σ***^2^
(ii) ***μ***_i_ are i.i.d across cross-sections with *E*(***μ***_i_) = 0, Var(***μ***_i_) = ***σ***^2^.
(iii) The initial observation satisfy $y_{i1}=\mu i/1-\emptyset +w_{i1}$ for i = 1, … … N

whereas wi1 $\sum_{j=0}^{\infty }ɸjs_{\text{it}}1-j$ and independent of ***μ***_i_

Blundell and Bond [80] provide the foundation for assumptions (i) and (ii), whereas Alvarez and Arellano [81] provide the foundation for assumption (iii). The following equation (2) shows the parameters of the dynamic model:

$${\Delta CO2}_{\text{it}}=\left(1-\gamma \right){CO2}_{it-1}+\alpha_{1}{\text{TECT}}_{\text{it}}+\alpha_{2}{\text{TOP}}_{\text{it}}+\alpha_{31VIDCOdent\;of\;uin\;satisfy\;oces\;the\;y\;demand\;er\;capita,trade\;opness\;nd\;installing\;ernergy}{\text{REC}}_{\text{it}}{+\alpha }_{4}{\text{IND}}_{\text{it}}$$

(2) $$+\alpha_{5}{\text{RGDP}}_{\text{it}}+\alpha_{6}{D\_\text{COVID}19}_{\text{it}}+\varepsilon_{\text{it}}\ldots \ldots ..$$

Econometric modeling uses a GMM estimator for parameter estimates. Dynamic panel data mixes time series with cross-sectional observations, and this technique excels. DGMM estimators are used to estimating dynamic panel models using differenced data. DGMM estimators excel in scenarios with few periods and plenty of cross-sectional data. Small time constraints are prevalent in this research since gathering adequate time series data for each unit in a dynamic panel is challenging. The DGMM estimator helps handle missing variable endogeneity and bias in the circumstances. DGMM excels where N is the number of cross-sectional units. "Large N″ issues arise when cross-sectional units surpass periods. The DGMM estimator solves dynamic panel data analysis' cross-sectional dependence issue. The DGMM estimator will help analyze dynamic panel data with limited time series and plenty of cross-sectional information. It offers a scalable and fast method for estimating dynamic panel models, which may help with small T and large N.

The given estimators, including the differenced panel GMM estimator, quantile moments estimation, and cointegration panel estimators (FMOLS and DOLS), have distinct characteristics and serve different purposes in panel data analysis. The differenced panel GMM estimator is well-suited for capturing dynamic relationships and controlling for time-invariant unobserved heterogeneity. It is useful for tracking shifts through time and isolating the effects of isolated variables because of the differencing technique it employs to deal with the possibility of endogeneity. However, quantile moments estimate considers variation in the conditional distribution at various quantiles. It helps study various impacts and determine the factors that affect various areas of the distribution since it reveals how the connection between variables changes throughout the distribution. Long-run correlations and co-movement between variables may be examined with the help of cointegration panel estimators like FMOLS and DOLS. These estimators assume the long-run equilibrium, endogeneity, and serial correlation are also dealt with it. They shine brightest when evaluating the interdependencies among variables across time and when researching economic phenomena with long-term dynamics.

The differenced GMM estimator has various benefits and drawbacks when used in panel situations. It effectively uses panel data, which is one of its main advantages. Controlling for endogeneity and unobserved heterogeneity, the estimator uses variable differentiation. The panel's ability to estimate cross-time and cross-entity links between variables is improved. When there are unobserved individual-specific effects or correlated mistakes, the differenced GMM estimator provides accurate estimates because of its consistency. Potential biases are reduced by incorporating individual-level fixed effects and correcting heteroskedasticity and serial correlation in the errors. The estimator's adaptability to dynamic models makes it a good fit for studying the evolution of connections over time. The constraints of the differenced GMM estimator should be noticed, however. Depending on the choice of moment conditions, lag structure, and instrument validity, it might be sensitive to the differenced variables, which it believes to be exogenous. To get accurate numbers, a high sample size is also necessary. When using the differenced GMM estimator in panel settings, it is important to remember these things.

The panel Granger causality test was applied on the given data set and computed F-statistics value to get different plausible outcomes.

i) **One-Way Causality: CO2** Granger causes TECT, REC, TOP, IND, RGDP, and D_COVID19 but not vice versa.
ii) **Reverse Causality:** TECT, REC, TOP, IND, RGDP, and D_COVID19 Granger cause CO2 emissions but not vice versa.
iii) **Feedback Linkage:** The bidirectional casual relationships exists between the variables, and
iv) **Neutrality:** The given variables do not have any cause-effect relationships although highly correlated.

Equation (3) shows the VAR Granger formulation:

(3) $$\left[\begin{array}{l} \ln\;{\left(CO2\right)}_{t}\\ \ln\;{\left(TECT\right)}_{t}\\ \ln\;{\left(REC\right)}_{t}\\ \ln\;{\left(TOP\right)}_{t}\\ \ln\;{\left(IND\right)}_{t}\\ \ln\;{\left(RGDP\right)}_{t}\\ D\_COVID19 \end{array}\right]=\left[\begin{array}{l} \tau_{0}\\ \tau_{1}\\ \tau_{2}\\ \tau_{3}\\ \tau_{4}\\ \tau_{5}\\ \tau_{56} \end{array}\right]+\sum_{i=1}^{p}\left[\begin{array}{l} \sigma_{11t}\sigma_{12t}\sigma_{13t}\sigma_{14t}\sigma_{15t}\\ \sigma_{21t}\sigma_{22t}\sigma_{23t}\sigma_{24t}\sigma_{25t}\\ \sigma_{31t}\sigma_{32t}\sigma_{33t}\sigma_{34t}\sigma_{35t}\\ \sigma_{41t}\sigma_{42t}\sigma_{43t}\sigma_{44t}\sigma_{45t}\\ \sigma_{51t}\sigma_{52t}\sigma_{53t}\sigma_{54t}\sigma_{55t}\\ \sigma_{61t}\sigma_{62t}\sigma_{63t}\sigma_{64t}\sigma_{65t} \end{array}\right]\times \left[\begin{array}{l} \ln\;{\left(CO2\right)}_{t-1}\\ \ln\;{\left(TECT\right)}_{t-1}\\ \ln\;{\left(REC\right)}_{t-1}\\ \ln\;{\left(TOP\right)}_{t-1}\\ \ln\;{\left(IND\right)}_{t-1}\\ \ln\;{\left(RGDP\right)}_{t-1}\\ D\_COVID19 \end{array}\right]+\sum_{j=p+1}^{d\;\max}\left[\begin{array}{l} \theta_{11j}\theta_{12j}\theta_{13j}\theta_{14j}\theta_{15j}\\ \theta_{21j}\theta_{22j}\theta_{23j}\theta_{24j}\theta_{25j}\\ \theta_{31j}\theta_{32j}\theta_{33j}\theta_{34j}\theta_{35j}\\ \theta_{41j}\theta_{42j}\theta_{43j}\theta_{44j}\theta_{45j}\\ \theta_{51j}\theta_{52j}\theta_{53j}\theta_{54j}\theta_{55j}\\ \theta_{61j}\theta_{62j}\theta_{63j}\theta_{64j}\theta_{65j} \end{array}\right]\times \left[\begin{array}{l} \ln\;{\left(CO2\right)}_{t-j}\\ \ln\;{\left(TECT\right)}_{t-j}\\ \ln\;{\left(REC\right)}_{t-j}\\ \ln\;{\left(TOP\right)}_{t-j}\\ \ln\;{\left(IND\right)}_{t-j}\\ \ln\;{\left(RGDP\right)}_{t-j}\\ D\_COVID19 \end{array}\right]+\left[\begin{array}{l} \varepsilon_{1}\\ \varepsilon_{2}\\ \varepsilon_{3}\\ \varepsilon_{4}\\ \varepsilon_{5}\\ \varepsilon_{6}\\ \varepsilon_{7} \end{array}\right]$$

Equations (4)–(10) shows multivariate Granger causality. i.e.,

(4) $$CO2_{t}=c_{1}+{\sum_{i=1}^{2}\beta_{1}CO2}_{t-i}+\sum_{i=1}^{2}\beta_{2}TECT_{t-i}+\sum_{i=1}^{2}\beta_{3}REC_{t-i}+\sum_{i=1}^{2}\beta_{4}TOP_{t-i}+{\sum_{i=1}^{2}\beta_{5}IND}_{t-i}+{\sum_{i=1}^{2}\beta_{6}RGDP}_{t-i}+{\sum_{i=1}^{2}\beta_{6}D\_COVID19}_{t-i}+\varepsilon$$

(5) $$TECT_{t}=c_{1}+{\sum_{i=1}^{2}\beta_{1}TECT}_{t-i}+\sum_{i=1}^{2}\beta_{2}CO2_{t-i}+\sum_{i=1}^{2}\beta_{3}REC_{t-i}+\sum_{i=1}^{2}\beta_{4}TOP_{t-i}+{\sum_{i=1}^{2}\beta_{5}IND}_{t-i}+{\sum_{i=1}^{2}\beta_{6}RGDP}_{t-i}+{\sum_{i=1}^{2}\beta_{6}D\_COVID19}_{t-i}+\varepsilon$$

(6) $$REC_{t}=c_{1}+{\sum_{i=1}^{2}\beta_{1}REC}_{t-i}+\sum_{i=1}^{2}\beta_{2}TECT_{t-i}+\sum_{i=1}^{2}\beta_{3}CO2_{t-i}+\sum_{i=1}^{2}\beta_{4}TOP_{t-i}+{\sum_{i=1}^{2}\beta_{5}IND}_{t-i}+{\sum_{i=1}^{2}\beta_{6}RGDP}_{t-i}+{\sum_{i=1}^{2}\beta_{6}D\_COVID19}_{t-i}+\varepsilon$$

(7) $$TOP_{t}=c_{1}+{\sum_{i=1}^{2}\beta_{1}TOP}_{t-i}+\sum_{i=1}^{2}\beta_{2}TECT_{t-i}+\sum_{i=1}^{2}\beta_{3}REC_{t-i}+{\sum_{i=1}^{2}\beta_{4}CO2}_{t-i}+{\sum_{i=1}^{2}\beta_{5}IND}_{t-i}+{\sum_{i=1}^{2}\beta_{6}RGDP}_{t-i}+{\sum_{i=1}^{2}\beta_{6}D\_COVID19}_{t-i}+\varepsilon$$

(8) $$IND_{t}=c_{1}+{\sum_{i=1}^{2}\beta_{1}IND}_{t-i}+\sum_{i=1}^{2}\beta_{2}TECT_{t-i}+\sum_{i=1}^{2}\beta_{3}REC_{t-i}+\sum_{i=1}^{2}\beta_{4}TOP_{t-i}+{\sum_{i=1}^{2}\beta_{5}CO2}_{t-i}+{\sum_{i=1}^{2}\beta_{6}RGDP}_{t-i}+{\sum_{i=1}^{2}\beta_{6}D\_COVID19}_{t-i}+\varepsilon$$

(9) $$RGDP_{t}=c_{1}+{\sum_{i=1}^{2}\beta_{1}RGP}_{t-i}+\sum_{i=1}^{2}\beta_{2}TECT_{t-i}+\sum_{i=1}^{2}\beta_{3}REC_{t-i}+\sum_{i=1}^{2}\beta_{4}TOP_{t-i}+{\sum_{i=1}^{2}\beta_{5}IND}_{t-i}+{\sum_{i=1}^{2}\beta_{6}CO2}_{t-i}+{\sum_{i=1}^{2}\beta_{6}D\_COVID19}_{t-i}+\varepsilon$$

(10) $$D\_COVID19_{t}=c_{1}+{\sum_{i=1}^{2}\beta_{1}D\_COVID19}_{t-i}+\sum_{i=1}^{2}\beta_{2}TECT_{t-i}+\sum_{i=1}^{2}\beta_{3}REC_{t-i}+\sum_{i=1}^{2}\beta_{4}TOP_{t-i}+{\sum_{i=1}^{2}\beta_{5}IND}_{t-i}+{\sum_{i=1}^{2}\beta_{6}RGDP}_{t-i}+{\sum_{i=1}^{2}\beta_{6}CO2}_{t-i}{+}_{t-i}+\varepsilon$$

The relationship between variables over time may be evaluated with the use of the variance decomposition analysis (VDA). Forecasting time series sometimes uses a statistical method called VDA, which dissects the overall variance of a time series into its component pieces to reveal the true causes of the series' inherent unpredictability. Essential phases in VDA include.

• Use a suitable approach for modelling the time series (e.g. ARIMA or State Space Model)
• Break down the residual variance into its separate parts, such as:
- Long-term shifts in the time series account for the "trend component," or the variance in the series.
- Seasonal Variability: Dispersion that arises from annual cycles (e.g. monthly patterns)
- The variation that arises from trends over a more extended period is referred to as the "cyclical component" (e.g. business cycles), and
- Unpredictable, or random, variation is referred to as the "Residual Component."
• Consider the influence of each factor on the prediction by analyzing their proportional share of the overall variance.

Understanding the numerous sources of variability in the data is essential to making accurate predictions, and variance decomposition analysis is a valuable tool for isolating the most important ones in a given time series.

## 5 Results

Table 1 shows descriptive data and a correlation matrix. Carbon emissions vary from 15.330 to 1.692 tonnes per capita yearly, with a standard deviation of 2.755 and a steeply skewed distribution with substantial kurtosis. Technological innovation in the form of patent applications averages 47785 and has a maximum value of 3459.395. Patent applications surpassed the variable's mean value of 829567 with a higher standard deviation. This variable is favorably skewed and peaks. The industry contributes 23.718 to GDP on average. On average, trade openness accounts for 110.847% of GDP and total energy use 23.894%. Regional per capita income and COVID-19 dummy are averaged by US$5.43E+11 and 0.600, respectively.

**Table 1:** Descriptive statistics and correlation matrix.

Methods	CO2	TECT	TOP	REC	IND	RGDP	D_COVID19
Mean	6.170	3459.395	110.847	23.894	23.718	5.43E+11	0.600
Maximum	15.330	47785	388.847	81.070	52.250	3.60E+12	1
Minimum	1.692	1	45.961	1.620	10.818	2.67E+09	0
Std. Dev.	2.755	8295.679	57.155	16.792	7.165	8.51E+11	0.491
Skewness	1.156	3.735	2.633	1.296	0.862	2.192	−0.408
Kurtosis	4.756	17.827	12.211	4.811	4.850	7.091	1.166
**Correlation Matrix**							
**Probability**	**CO2**	**TECT**	**TOP**	**REC**	**IND**	**RGDP**	**D_COVID19**
CO2	1						
	–						
TECT	0.208	1					
	(0.003)	–					
TOP	0.469	−0.258	1				
	(0.000)	(0.000)	–				
REC	−0.299	−0.227	−0.138	1			
	(0.000)	(0.001)	(0.053)	–			
IND	0.027	0.092	−0.163	−0.213	1		
	(0.704)	(0.197)	(0.022)	(0.002)	–		
RGDP	0.087	0.840	−0.303	−0.258	−0.037	1	
	(0.224)	(0.000)	(0.000)	(0.000)	(0.600)	–	
D_COVID19	−0.050	−0.009	−0.006	0.011	−0.004	0.002	1
	(0.479)	(0.894)	(0.925)	(0.871)	(0.946)	(0.969)	–

The correlation matrix demonstrates that green energy efforts are inversely correlated with carbon emissions, showing that the regional decarbonization objective may be realized by growing REC across nations. Technology innovation, trade openness, and industrial value-added positively affect carbon emissions, and regional economic expansion favorably contributes to technological innovation. Table 2 shows the first and second generation panel unit root tests for ready reference.

**Table 2:** First and second generation unit root estimates.

Variables	IPS Unit Root Estimates			
	Level	Prob.value	First Difference	Prob. value
CO2	−2.729*	0.069	−6.324***	0.000
IND	−0.606	0.869	−3.271***	0.015
REC	−2.442	0.131	−3.486***	0.007
RGDP	−3.459***	0.009	−5.843***	0.000
TECT	−2.117	0.236	−3.903***	0.002
TOP	−2.342	0.160	−5.982***	0.000
CADF Unit Root Estimates				
CO2	−1.811	0.377	−4.998***	0.001
IND	−1.456	0.558	−5.507***	0.000
REC	−1.622	0.478	−4.112***	0.001
RGDP	−1.755	0.402	−2.813*	0.057
TECT	−1.682	0.450	−4.466***	0.000
TOP	−1.895	0.336	−6.020***	0.000

The findings from the IPS unit root estimates indicate that CO2 emissions and RGDP display stationary levels, while the other variables exhibit first-differenced stationarity. Conversely, the CADF (second generation) unit root estimates confirm that all variables possess an order of integration of one, suggesting they follow a differenced stationary series. These results highlight the differing properties of the variables under consideration and provide insights into their long-term and short-term dynamics. Table 3 shows the cross-sectional dependence (CSD) estimates.

**Table 3:** CSD test estimates.

Variables	Breusch-Pagan LM	Prob. value
CO2	2597.858***	0.000
TECT	1303.160***	0.000
REC	2209.169***	0.000
RGDP	2114.746***	0.000
IND	1274.620***	0.000
TOP	2109.346***	0.000
D_COVID19	3705.121***	0.000

The results show that the observations within a cross-section are not independent of each other, violating one of the key assumptions of classical panel models. This dependence can arise due to various reasons, such as common unobserved factors, spatial proximity, or interactions among the individuals in the panel. Table 4 displays the Panel Dynamic GMM model results, which demonstrate a positive relationship between ecological sustainability and carbon emissions across a panel of ECA countries, with emissions increasing in line with the level of circular economy activity. The current study's findings are supported by the previous research of Mentel et al. [82], Du et al. [83], Anser et al. [84], and Fuinhas et al. [85], whom all agree that developing REC to create a stable investment foundation and new environmental initiatives is essential. Furthermore, each nation may establish a local REC deployment plan that describes the primary objective of replacing carbon energy with clean energy and environmental sustainability. Regional integration has to support the REC technology innovation infrastructure to find a new REC source and supply clean energy implementation, which helps decrease carbon emissions.

**Table 4:** Differenced panel GMM, Granger causality and variance decomposition estimates.

Variables	PDGMM-1		PDGMM-2	PDGMM-3	PDGMM-4	PDGMM-5
CO2_t-1_	0.033 (0.191)		−0.083** (0.028)	0.170*** (0.000)	0.026* (0.070)	0.042 (0.091)
IND	0.031 (0.443)		0.103 (0.253)	0.075** (0.016)	–	0.029 (0.127)
REC	−0.668*** (0.000)		–	–	0.685*** (0.000)	0.737*** (0.0000)
RGDP	−0.038 (0.528)		−0.440** (0.045)	–	−0.0002 (0.904)	−0.022 (0.107)
TECT	−0.011 (0.282)		0.036*** (0.0060)	–	−0.009 (0.492)	–
TOP	0.017 (0.571)		0.184 (0.103)	−0.039*** (0.009)	–	–
D_COVID19	−0.013** (0.028)		–	−0.0211*** (0.010)	0.010* (0.075)	−0.0145** (0.013)
**Statistics Tests**						
J-statistics	3.829 (0.574)		13.181 (0.021)	4.3190 (0.504)	4.127 (0.659)	4.023 (0.545)
Arellano-Bond Serial Correlation Test(Prob)	AR(1)	0.458	0.196	0.189	0.406	0.507
	AR(2)	0.656	0.488	0.466	0.491	0.795
**Panel Granger Causality Estimates**						
TECT↔IND	REC→CO2		CO2→RGDP, TECT, and TOP		TOP and IND→ RGDP	
RGDP→REC and TECT			TECT→REC			
**VDA Estimates (2023 to 2031)**						
TECT influenced 5.316% of carbon emissions			TOP Influenced 2.511% of carbon emissions		REC influenced 0.843% of carbon emissions	

There is an inverse link between the use of renewables and carbon emissions, which indicates that regional green activities effectively reduce carbon emissions. The findings of this study, in conjunction with those of previous research conducted by Rasheed et al. [86], Usman et al. [87], Ponce & Khan [88], and Piłatowska & Geise [89]. The studies indicated that it was necessary to create a particular threshold limit and call for flexible energy production and consumption solutions, including using subsidies for biogas and hydropower producers in certain nations. Introduce and enact new legislative policies to subsidies green energy, provide low-interest loans and subsidies to businesses and people, and encourage the widespread use of REC technologies for sustainable outcomes.

The fact that economic growth slows down in areas with high carbon emissions demonstrates the importance of regional cooperation in preserving the planet's biosphere and advancing the decarbonization agenda, both of which are essential for bringing about the kind of widespread prosperity that benefits everyone. Previous research conducted by Sharif et al. [90], Elfaki et al. [91], Bekun et al. [92], and Mohsin et al. [93] lends credence to the findings of the present study. It suggests that a rise in the share of REC in the conventional energy mix, encouragement of the use of clean energy assets, and discouragement of dependency on energy derived from fossil fuels should be implemented. In addition, there is an immediate need to create conversion the production of fossil fuels towards the production of clean energy in order to accomplish the applicable climate objectives eventually.

Global carbon output is positively correlated with embedded technology emissions. Regional economic integration is inversely correlated with carbon emissions, supporting that freer commerce spreads ecologically benign ideas. This study and others by Adebayo et al. [94], Dong et al. [95], and Wang et al. [96] show that low-carbon economy measures, REC promotion, and technological innovation financing can lead to sustainable development. The COVID-19 dummy reduces nations' susceptibility to coronavirus illness and reduces carbon emissions. Ray et al. [97], Liu et al. [98], and Sarfraz et al. [99] have underlined the necessity to switch to REC sources and cleaner energy technology. Ecological sustainability requires economic sectors to employ green resources and maintain the environment for all life. Increase national development in carbon emission trading's miracle technology.

These results suggest a positive link between regional carbon emissions and industry value addition and knowledge transfer. This makes logical because both require energy-intensive industrial processes that emit GHGs. Renewables, regional development, and easier trade reduce carbon emissions. Renewables have lower carbon footprints than conventional ones, and a strong economy and unrestrained commerce may spur innovation and investment in green technologies [100]. Policy implications include that industrial development and technological transfer without environmental consideration may not reduce carbon emissions. Policies to promote renewables, regional economies, and international trade might cut carbon emissions. These results support renewables regulations, ecologically responsible economic growth, and global markets for greener goods [101]. The results also suggest measures that balance economic development and environmental conservation. Such regulatory initiatives encourage cleaner technologies, green funding, and sustainable infrastructure. These policies enable investments in greener technologies and accelerate the transition to a sustainable future [102].

Granger causality estimations confirmed the following cause-and-effect connections that support sustainable policies.

I. Regional growth and high carbon emissions hampered clean and green development.
II. Emissions fueled regional technology developments that weaken environmental sustainability and increase global warming risk.
III. Emissions increase polluting goods trade, confirming the pollution haven hypothesis.
IV. Trade and industrial output create economies of scale.
V. Economic development increases demand for renewables and improves technical infrastructure. Growth areas lead this endeavour.
VI. As technology progresses, more countries establish green energy initiatives,
VII. Technology boosts industrial productivity and eco-friendly production.

According to the VDA results, it is possible to foresee the influence of technological advancements, increased international commerce, and the pursuit of green energy on carbon dioxide emissions during the next decade. The study indicates that technology has the highest predicted influence, accounting for 5.316% of the difference in carbon emissions, followed by trade openness, which is expected to account for 2.511%. In terms of its effect on carbon emissions, the push toward renewable power is expected to be relatively minor. This research shows that these three criteria are crucial to cutting down on carbon output and fostering a more sustainable future. Governments and organizations may help reduce carbon emissions and adapt to climate change by prioritizing the creation of cleaner technology, advocating for freer trade, and fostering the use of green energy. The necessity for regional economic integration and investment in REC sources may pave the road to growth and prosperity. Table 5 shows the robustness tests, i.e., quantiles regression, panel FMOLS, and DOLS for ready reference.

**Table 5:** Quantile regression, FMOLS, and DOLS estimates.

Variables	Quantile Regression	FMOLS	DOLS
TECT	9.83E-05***	0.0001**	0.0001
REC	−0.022*	−0.040**	−0.045*
RGDP	−3.06E-13	−9.28E-13	−7.55E-13
IND	−0.064**	0.014	0.005
TOP	0.023***	0.019***	0.020***
D_COVID19	−0.324	−0.245	−0.634
Constant	5.266***	3.022**	3.640
**Statistical Tests**			
R^2^	0.171	0.406	0.445
Quasi LR Test	49.616***	–	–

Using panel quantile regression and the FMOLS estimator, the study found strong evidence of a correlation between regional technology transfer and carbon emissions. These results demonstrate that emissions associated with the use of technology occur in all countries. In addition, there is a negative association between REC and carbon emissions across all three estimators, demonstrating that the transition to green energy is crucial to accomplishing the decarbonization objective. Carbon emissions were shown to be considerably affected by both industrial value addition and trade openness. While it was found that environmentally friendly manufacturing methods helped lower carbon emissions, it was also shown that freer commerce might be harmful to the planet. Therefore, creating and maintaining economic policies that give equal weight to fostering prosperity and protecting the natural world is crucial. When compared to the differenced GMM estimations, the obtained findings are reliable. This means that the estimated model with differenced GMM does a good job of capturing the interrelationships between the relevant variables. The Sargan-Hansen J-statistics and the variance inflation factor (VIF) are calculated in Table 6 to determine whether or not the moment the GMM estimator meets the criteria and checks for multicollinearity. First, the model is estimated under moment circumstances in the GMM framework, and then the over-identifying limitations are tested by comparing the estimated values with the observed ones. The Sargan-Hansen J-statistics is found by subtracting the theoretical value from the observed one.

**Table 6:** First and second step differenced GMM's Sargan-Hansen statistics and VIF value.

	J-statistics	Sargan-Hansen J-statistics
First Step	0.656	0.985
**Second Step**	**1.717**	**0.886**
**VIF Value**		
**Multicollinearity Test Estimates**	TECT	3.52
	REC	1.252
	RGDP	3.843
	IND	1.189
	TOP	1.245
	DUM	1.002
	Constant	–

The findings indicate that the lack of significance in both the first and second steps of the Sargan-Hansen test suggests the validity of the instrumental variables used in the analysis. This implies that the chosen instrumental variables effectively address any potential endogeneity concerns and that the model specification accurately represents the relationship between the variables of interest. Moreover, the Variance Inflation Factor (VIF) values, which measure multicollinearity, remain below the threshold value of 10. Therefore, based on this observation, it is safe to conclude that the model is devoid of any significant multicollinearity issues.

## 6 Discussion

Findings from this research indicate a positive link between industrial ecology and carbon emissions across a sample of ECA countries. The research shows that the circular economy's intensity correlates with an increase in emissions. This data may indicate a causal link between the development of the circular economy and the release of greenhouse gases. This finding has implications because it prompts policymakers to weigh the benefits of a circular economy against the costs of lowering carbon emissions [103]. The increasing production and consumption that often follow the expansion of the circular economy may account for this observation [104]. Increased product and service consumption might lead to higher energy demands and greater GHG emissions if companies and consumers increasingly embrace circular models [105,106]. The use of conventional energy sources like fossil fuels in manufacturing circular economy products is another probable cause. Despite the circular economy's goal of reducing waste and encouraging the use of more environmentally friendly products, this may lead to more emissions. These results highlight the need for more study into the connection between industrial ecology and carbon emissions in this setting and emphasize the need to consider the circular economy's economic and environmental consequences [107,108].

The adoption of regional green activities may positively influence decreasing emissions, as the discovery that there is an inverse link between the REC and carbon emissions reveals. The reason(s) behind this might be varied. First, switching to REC reduces reliance on fossil fuels, the most common cause of GHG emissions. The move away from fossil fuels and towards low-carbon alternatives may be further encouraged by the development of clean energy infrastructure and technologies facilitated by the widespread use of green energy sources [30]. A further reduction in carbon emissions may result from increased usage of sustainable power since it can stimulate the development of energy-efficient technology and practices. Regional green activities may successfully lower emissions and contribute to a sustainable future, as shown by the inverse link between REC and carbon emissions [109].

The negative link between economic expansion and high carbon emissions highlights the importance of regional collaboration in attaining environmental sustainability and economic development. High carbon emissions are considered a barrier to economic progress, highlighting the need for decarbonization activities in these regions. To reduce carbon emissions and protect the biosphere, a worldwide and regional effort to work together is essential, as is the conclusion reached here. Taking this tack may speed up the decarbonization process, essential to fostering long-term, mutually beneficial economic growth [110,111].

Multiple explanations might be offered for the positive relationship between technological development and carbon emission. Nations' manufacturing activities often expand with technological development, increasing carbon emissions. Moreover, nations can create and consume more thanks to technological developments, driving up energy demand and, in turn, carbon emissions [112]. In addition to increasing carbon emissions, technological development drives more urbanization and industrialization. That is why nations need to prioritize sustainable development, balancing advancing technology and protecting the environment [113].

A negative relationship between regional trade integration and carbon emissions shows that freer trade may facilitate the spread of environmentally friendly innovations. Several factors may contribute to this phenomenon.

• When nations are allowed to trade more freely with one another, breakthrough technologies that aid in sustainable development may be more easily shared and accessed [114].
• Nations may be better able to work together to create effective environmental legislation and share best practices if they are economically integrated [115].
• New markets might increase demand for eco-friendly products, which could motivate companies to fund R&D [116].

Taken together, these elements may strengthen the decarbonization goal and aid in reducing carbon emissions.

Possible explanations for the inverse relationship between the COVID-19 dummy and carbon emissions include a drop in economic activity and energy use due to the pandemic. Because of lockdowns and travel restrictions imposed because of the epidemic, many companies and factories had to temporarily close down, resulting in a decrease in energy use. There may have been a move to remote work due to the epidemic, which would also have reduced in-office activity and energy demand [117]. However, the decline in carbon emissions may reflect a temporary slowdown in economic activity rather than a genuine transition to greener energy. To know the entire extent of the pandemic's effect on carbon emissions and the global energy system, further study is required [118].

## 7 Conclusions

This study delves into the intricate dynamics of sustainable development, analyzing the trade-offs involved in transitioning to clean energy sources. The study aims to shed light on the challenges and opportunities for achieving environmentally friendly economic growth in a panel of 39 ECA countries. The study employed multiple estimators, such as two-step GMM, quantile regression, and cointegration panel analysis. These estimators provided a comprehensive analysis of the research variables, assessing moment conditions, conditional relationships, and long-term equilibrium relationships from different perspectives. The findings may help cut carbon emissions. Industrial value-added and knowledge transfers increase carbon emissions, whereas renewables, regional economic development, and trade openness decrease them. To minimize carbon emissions, governments should promote clean energy and renewable sources. Tax concessions and subsidies encourage private enterprises to create REC infrastructure. Our results suggest that regional economic growth and trade openness reduce carbon emissions, and policymakers should prioritize them. Government investments in education, infrastructure, and job-creating enterprises may boost regional economic growth. Eliminating trade barriers and boosting international cooperation and trade agreements prioritizing environmental protection would boost commerce. Since industries and technology transfer increase regional carbon emissions, authorities should support sustainable production techniques and oppose polluting technologies. Carbon taxes or cap-and-trade policies may encourage companies to reduce carbon emissions and invest in green technologies.

Policy implications derived from the Granger causality results offer constructive suggestions for reducing carbon emissions and promoting long-term economic development. Firstly, policies should facilitate the transfer of environmentally friendly technology to companies and encourage the adoption of sustainable practices in the industry. This can be achieved by promoting cooperation between businesses and academic institutions, and providing tax incentives or subsidies for the use of green technology. Secondly, stimulating the adoption of REC sources is crucial, which can be supported through subsidies for REC projects, increased funding for renewables R&D, and the implementation of policies promoting energy efficiency and conservation. Thirdly, to foster sustainable economic growth while reducing carbon emissions, policies should encourage sustainable mobility and investments in low-carbon infrastructure. Implementing rules to incentivize the adoption of sustainable practices by companies and families can further drive adoption. Fourthly, policies that promote free trade while supporting the transfer of environmentally friendly technology can contribute to sustainable economic development. This can be achieved through international trade agreements that encourage the dissemination of green technology and responsible corporate practices. Lastly, policies encouraging sustainable economic development can have positive environmental impacts by funding educational and training initiatives that promote sustainable practices and technology adoption. The following economic, environmental and societal policy impacts are as follows.

- Economic Policy Impacts:
  • Governments should promote clean energy and renewable sources by providing tax concessions and subsidies for private enterprises to develop REC infrastructure.
  • Government investments in education, infrastructure, and job-creating enterprises can boost regional economic growth and contribute to long-term development.
  • Policies that encourage sustainable economic growth while simultaneously lowering carbon emissions should be implemented. This can be achieved through measures such as promoting sustainable mobility and investing in low-carbon infrastructure.
  • Introducing rules and regulations may facilitate businesses' and households' broad adoption of sustainable practices.
- Environmental Policy Impacts:
  • The business sector must embrace sustainable practices and implement policies that facilitate the transfer of environmentally friendly technologies to corporations. To this end, it is possible to provide tax incentives or subsidies for the adoption of green technology and to encourage collaboration between enterprises and academic institutions.
  • Stimulating the adoption and REC sources is crucial. Governments should consider providing subsidies for REC projects and increasing funding for green R&D. Energy efficiency and conservation policies should also be encouraged.
- Societal Policy Impacts:
  • Sustainable economic growth may be aided by policies promoting free trade and facilitating the transfer of environmentally friendly technologies. International trade agreements that promote the dissemination of environmentally friendly technology and the adoption of responsible corporate practices should be pursued.
  • Encouraging sustainable economic development can positively affect the environment. Government regulations can fund educational and training initiatives that encourage firms and individuals to embrace sustainable practices and technology.

By implementing carbon pricing, companies may be incentivized to minimize carbon emissions and invest in green technology. Authorities should support sustainable production techniques and oppose polluting technologies to minimize regional carbon emissions. By implementing these comprehensive policies, governments can effectively address the economic, environmental, and societal aspects of carbon emissions, promoting sustainable development while mitigating the negative impacts on the environment.

The study's findings may suggest further research and limitations. Due to its focus on higher-income ECA nations, this study's findings may not generalize to other regions or lower-income countries. Future research should study how regional economic integration influences carbon reduction in wide regions and socioeconomic levels. Since the study only covered 2017–2021, the long-term effects of regional economic integration on carbon emissions are unidentified. Regional economic integration's long-term implications on carbon emissions may be studied. Even though regional green energy initiatives and sustainable technology transfers may cut carbon emissions, the study might have focused on policies and techniques that encourage these activities. Future research may examine the best regional strategies for expanding green energy projects and sustainable technology transfers. Finally, industrial output increases carbon pollution, highlighting the need for more research into strategies to reduce industrial emissions and promote sustainable industrial practices.

Regional economic integration and cooperation may help lessen the impact of climate change. By enforcing policies and launching programmes that encourage REC, technical advancement, and international commerce, we can ensure continued economic progress while minimizing the detriment brought on by carbon emissions. The ECA countries may help themselves and the globe by adopting these policies.

## Author contribution statement

Qiyun Zhou; Khalid Zaman: Analyzed and interpreted the data; Contributed reagents, materials, analysis tools or data; Wrote the paper.

Jianpeng Wu: Conceived and designed the experiments; Analyzed and interpreted the data; Wrote the paper.

Muhammad Imran: Conceived and designed the experiments; Contributed reagents, materials, analysis tools or data; Wrote the paper.

Abdelmohsen A. Nassani; Rima H. Binsaeed: Performed the experiments; Analyzed and interpreted the data; Contributed reagents, materials, analysis tools or data; Wrote the paper.

## Data availability statement

Data will be made available on request.

## Declaration of competing interest

The authors declare that they have no known competing financial interests or personal relationships that could have appeared to influence the work reported in this paper.
